# Arthritis suppression by NADPH activation operates through an interferon-β pathway

**DOI:** 10.1186/1741-7007-5-19

**Published:** 2007-05-09

**Authors:** Peter Olofsson, Annika Nerstedt, Malin Hultqvist, Elisabeth C Nilsson, Sofia Andersson, Anna Bergelin, Rikard Holmdahl

**Affiliations:** 1 Biovitrum AB, Arvid Wallgrens Backe 20, SE-413 46 Göteborg, Sweden; 2 Medical Inflammation Research, I11 BMC, Lund University, SE-221 84 Lund, Sweden

## Abstract

**Background:**

A polymorphism in the activating component of the nicotinamide adenine dinucleotide phosphate (NADPH) oxidase complex, neutrophil cytosolic factor 1 (NCF1), has previously been identified as a regulator of arthritis severity in mice and rats. This discovery resulted in a search for NADPH oxidase-activating substances as a potential new approach to treat autoimmune disorders such as rheumatoid arthritis (RA). We have recently shown that compounds inducing NCF1-dependent oxidative burst, e.g. phytol, have a strong ameliorating effect on arthritis in rats. However, the underlying molecular mechanism is still not clearly understood. The aim of this study was to use gene-expression profiling to understand the protective effect against arthritis of activation of NADPH oxidase in the immune system.

**Results:**

Subcutaneous administration of phytol leads to an accumulation of the compound in the inguinal lymph nodes, with peak levels being reached approximately 10 days after administration. Hence, global gene-expression profiling on inguinal lymph nodes was performed 10 days after the induction of pristane-induced arthritis (PIA) and phytol administration. The differentially expressed genes could be divided into two pathways, consisting of genes regulated by different interferons. IFN-γ regulated the pathway associated with arthritis development, whereas IFN-β regulated the pathway associated with disease protection through phytol. Importantly, these two molecular pathways were also confirmed to differentiate between the arthritis-susceptible dark agouti (DA) rat, (with an *Ncf-1*^*DA *^allele that allows only low oxidative burst), and the arthritis-protected DA.*Ncf-1*^E3 ^rat (with an *Ncf1*^E3 ^allele that allows a stronger oxidative burst).

**Conclusion:**

Naturally occurring genetic polymorphisms in the *Ncf-1 *gene modulate the activity of the NADPH oxidase complex, which strongly regulates the severity of arthritis. We now show that the *Ncf-1 *allele that enhances oxidative burst and protects against arthritis is operating through an IFN-β-associated pathway, whereas the arthritis-driving allele operates through an IFN-γ-associated pathway. Treatment of arthritis-susceptible rats with an NADPH oxidase-activating substance, phytol, protects against arthritis. Interestingly, the treatment led to a restoration of the oxidative-burst effect and induction of a strikingly similar IFN-β-dependent pathway, as seen with the disease-protective *Ncf1 *polymorphism.

## Background

Rheumatoid arthritis (RA) is one of the commonest autoimmune diseases, with a prevalence of 0.5–1% [1,2]. RA is a chronic and severely disabling disease of unknown etiology, although both environmental [3] and genetic factors [4] are believed to play roles in its cause. There is presently no cure for RA, although a variety of different drugs is used to treat the symptoms. The most common treatments include disease-modifying antirheumatic drugs such as,methotrexate [5,6]. Other efficient antirheumatic drugs are the recently developed biological-response modifiers, such as tumor necrosis factor (TNF)-α blockers, which reduce both the established inflammation and joint destruction [7]. The drawback of these treatments is an increased risk of infections because the body's defense system is impaired. Other biological-response modifiers targeting other cytokines or costimulatory molecules are currently being clinically tested and evaluated [8]. Despite this range of antirheumatic drugs, there is still a large number of RA patients for whom none of these treatments is effective [9,10], thus making the development of new therapies essential.

Identification of new targets for development of antirheumatic drugs is hampered by the heterogeneity of RA and the complexity of its molecular pathology. However, recent efforts with linkage-association studies, both in human populations and in animal models, are now giving results in the form of successful identification of autoimmunity-regulating genes [11-15]. The combined results from such studies will eventually lead to a greater understanding of the molecular regulation of this complex disease and they present potential new targets for drug development.

*Neutrophil cytosolic factor 1 *(*Ncf1*), also known as *p47phox*, was one of the first single genes identified to regulate arthritis severity. This finding was a result of linkage analysis and positional cloning in an arthritis model in rats using a cross between the arthritis-susceptible dark agouti (DA) rat and the arthritis-resistant E3 strains DA.*Ncf1*^E3^([14,16]. NCF-1 is the activating component of the NADPH oxidase complex, which, upon activation, produces reactive oxygen species (ROS) [17]. We found that the dramatic increase in arthritis severity was caused by a decreased capacity of the nicotinamide adenine dinucleotide phosphate (NADPH) oxidase complex to produce ROS. Interestingly, the lower oxidative burst led to a reduction in cell membrane proteins and activation of autoreactive and arthritogenic T cells [18]. Thus, the identified *Ncf1 *polymorphism and its effect on the immune system by decreased ROS production was concluded to be associated with the regulation of arthritogenic CD4 T cells in the immune priming phase [14,19].

NADPH oxidase-activating substances such as phytol (3,7,11,15-tetramethyl-2-hexadecene-1-ol), were identified from studies performed on a human neutrophil cell line, and have subsequently been shown to be very efficacious in the treatment of arthritis in rats [20]. However, the mechanism through which these compounds act is still not completely understood.

The intention of this study was to obtain a molecular insight into the anti-inflammatory mechanism of NADPH oxidase activation by phytol in an experimental arthritis model in rats, the pristane-induced arthritis (PIA) model [21]. To analyze the *in vivo *effects of the NADPH oxidase activator phytol versus the effects of the arthritis-inducing compound pristane, global gene-expression profiling was performed. A biodistribution analysis of phytol was performed to determine which tissue and time point would be most relevant to investigate. Phytol was observed to accumulate slowly in inguinal lymph nodes, with peak levels being reached approximately 11 days after subcutaneous administration, which coincides with the onset period of arthritis. In the global gene-expression profiling, inguinal lymph nodes obtained 10 days after administration were analyzed. This analysis revealed several genes that were differentially expressed in rats with PIA compared with rats injected with phytol. A group of interesting genes was selected and verified by quantitative real-time PCR analysis in three further separate biological experiments, including a comparative study between the DA and DA.*Ncf1*^E3 ^congenic rats. The comparison between the two strains was aimed at determining whether the selected genes were differentially expressed in the arthritis-susceptible DA rat versus the arthritis-resistant congenic DA.*Ncf1*^E3 ^rat. Such data would indicate whether the NADPH oxidase-activating compound phytol is activating a pathway that is normally inactive in the absence of a fully functional *Ncf1*.

By studying gene-expression profiles and performing pathway analysis, we have identified the importance of an IFN-β-dependent pathway as one molecular mechanism for the arthritis-ameliorating efficacy of NADPH oxidase-activating compounds.

## Results

The discovery that NADPH oxidase-derived ROS have disease-protecting effects in arthritis opens up new possibilities for drug development against autoimmune and inflammatory diseases. To obtain a more detailed understanding of the molecular mechanism of this regulation, gene-expression profiling experiments were performed on inguinal lymph nodes from rats with pharmacologically (phytol) and genetically (*Ncf1*) modified NADPH oxidase activity.

### Biodistribution and NADPH oxidase activation

**Figure 1:**
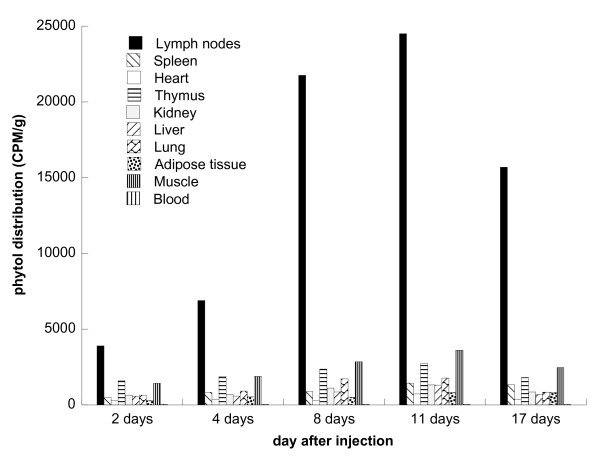
**Tissue distribution of tritium-labeled phytol in rats. Biodistribution was estimated as relative counts per minute per gram of tissue**. A large fraction of phytol remained as a depot in the injection site (> 90%, not shown). Besides that, the inguinal lymph nodes were the primary tissue for accumulation of phytol. The distribution of phytol to the inguinal lymph nodes showed the highest accumulation > 1 week after injection and showed a reduction after 2 weeks. Values are means from groups of four animals.

Phytol has previously been identified as a potent NADPH oxidase activator [14,20]. The *in vivo *distribution of phytol was analyzed and examined by subcutaneous injection of tritium-labeled phytol into the base of the tail in DA rats. The rats were killed and dissected 2, 4, 8, 11 and 17 days after administration. Various organs (inguinal lymph nodes, spleen, heart, thymus, kidney, liver, lung, fat, muscle and peripheral blood) were collected, homogenized and analyzed in a beta counter to determine tissue-incorporated tritiated phytol. Most of the administered phytol remained as a depot at the injection site (> 90%, data not shown), whereas phytol distributed to tissues was recovered mainly from the inguinal lymph nodes. Furthermore, the accumulation of phytol in the lymph nodes reached its maximum level 11 days after administration (Figure 1). No phytol was detected in peripheral blood at the time points studied or in blood collected at earlier time points (2, 6, 13 or 25 hours after administration, data not shown). Further analysis of the biodistribution of phytol at the microscopic level in the inguinal lymph nodes was performed by microautoradiography. It was shown that phytol was distributed primarily to the cortical regions and also in the sinusoidal space of the inguinal lymph nodes (data not shown). Moreover, the staining seemed to appear between cells or in the cell membrane, but not intracellularly.

### Global gene-expression profiling

**Figure 2:**
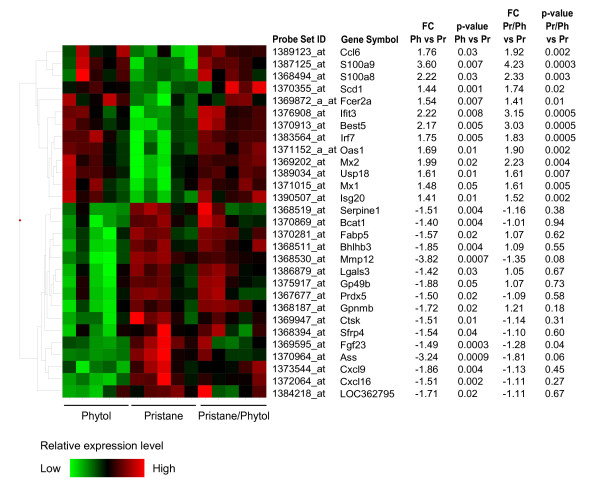
**Hierarchical clustering of differentially expressed genes in phytol-treated compared with pristane-treated rats**. Euclidean distance was used as a similarity measure. Each column represents one individual rat, and each horizontal stripe represents a gene transcript. The Affymetrix probe set identification, the gene symbol, the average fold change (FC) for phytol versus pristane together with the *p *value, and the average fold change for phytol/pristane versus pristane and its *p *value are given to the right. The colors in the clustering represent the gene-expression level in each individual rat compared with the average expression in all arrays, where green indicates low expression and red represents high expression.

Previous results have shown that the NADPH oxidase activator phytol has ameliorating properties on arthritis when administrated prior to arthritis induction and when given as therapeutic treatment [20]. To obtain a molecular understanding of the protective effects of phytol on PIA, four groups of animals were subjected to different conditions; (i) induced arthritis by injection with pristane, (ii) injection with phytol, (iii) injection with both pristane and phytol, and (iv) no treatment. All animals were killed close to disease onset, i.e.10 days after administration. Global gene-expression profiling using chip arrays (Genechip^®^;Affymetrix, Santa Clara, CA, USA) was performed on the collected inguinal lymph nodes from the described animals. The gene-expression pattern was quite different between treated rats and naïve controls. Comparing any of the group of treated animals and untreated animals, > 350 differentially expressed genes were detected (at significance level *p *< 0.05 and absolute fold change > 1.4; data not shown). However, as the most interesting differences were to be found between the different treatments, the analysis focused on these groups. When comparing phytol-treated versus pristane-treated animals, the expression of 29 genes was significantly changed (*p *< 0.05, with absolute fold change > 1.4). Of these 29 genes, 13 showed expression in phytol-treated rats, whereas 16 genes showed higher expression in pristane-treated rats (Figure 2). Because more complex gene induction was observed in animals given phytol plus pristane, further studies concentrated on analyses of single-compound administrations.

### Quantitative real-time PCR validation of differentially expressed genes

To minimize erroneous conclusions due to technical variability and multiple testing effects inherent to the microarray technology, as well as biological variation, quantitative real-time PCR analysis was used to validate expression profiles of nine differentially expressed genes (Table 1) in separate biological material. This verification confirmed all examined transcripts to be differentially expressed (data not shown). In addition, there was strong correlation between the fold changes detected using global gene expression and the quantitative real-time PCR analysis for the identified genes (Table 1; Pearson correlation coefficient R^2 ^> 0.96) [22].

#### The genes upregulated by injections with pristane were Ass, *Cxcl9 *and *Mmp12*

*Ass *(*argininosuccinate synthetase*) encodes an enzyme involved in the urea cycle mediating the condensation of citrulline and aspartic acid to form arginine. The expression of *Ass *is induced by common pro-inflammatory cytokines (interleukin (IL)-1β, interferon (IFN)-γ and TNF-α) [23,24]. CXCL9 (Chemokine (C–X–C motif) ligand 9, also known as Mig, (monokine induced by IFN-γ)) is a chemokine belonging to the CXC subfamily of chemokines acting through G-protein-coupled receptors [25]. These proteins have chemoattraction and growth-promoting properties and are induced by IFN-γ and, to some extent, TNF-α to attract T cells to sites of inflammation [26]. Higher levels of CXCL9 expression have been observed in the synovial tissues and fluids of patients with RA compared with tissues and fluids from control patients [27]. MMP12 (matrix metalloproteinase 12) belongs to the family of matrix metalloproteinases (MMPs) [28], which are involved in cell migration and degradation of cartilage and bone by remodeling the extracellular matrix. Inducers of expression of MMPs include IL-1β, TNF-α [29] and IFN-γ [30]. Abnormal levels of MMPs have been found in patients with autoimmune disorders such as RA and multiple sclerosis [31].

#### The genes upregulated by phytol were *Best5*, *Irf7*, *Ifit3*, *Oas1*, *Mx2 *and *S100a9*

Best5 (bone-expressed sequence tag 5) is mainly expressed in bone marrow and spleen and has been proposed to be involved in bone formation. The cytokines IFN-α and IFN-γ have been shown to induce BEST5 expression in osteoblasts [32].Interferon regulatory factor (IRF)-7 belongs to the IRF family of transcription factors involved in cell growth, antiviral defense and immune activation in lymphoid cells in spleen, thymus, and peripheral blood [33,34]. IRF7 is part of the pathway activated by viral infection to induce the production of IFN type I (IFNtα/β) by a positive feedback loop [35]. IRF7 was recently reported to be the master regulator of IFN type I-dependent immune responses [36], and recent publications have shown that an IRF5 polymorphism is strongly associated with systemic lupus erythematosus (SLE) [37,38]. Interferon-induced protein with tetratricopeptide repeats (IFIT)-3 is induced by IFN-α [39] and IFN-β but not IFN-γ [40]. However, IRF-7 most probably has a dual role, being both a strong regulator of IFN expression [35] and being induced by IFN in a positive feedback regulation [35,41].

The protein 2',5'-oligoadenylate synthetase (OAS)-1 belongs to the OAS family, which was one of the first groups of IFN-induced antiviral proteins to be characterized. The activation of this mechanism is induced by the IFN type I pathway, which is activated upon pathogenic invasion as part of an antiviral response [42].

Myxovirus resistance (MX)-2 has a functional role in the defense against viral infections and its expression is partly controlled by type I IFNs [43]. MX2 is induced by type I IFNs, and to a less extent by IFN-γ and lipopolysaccharide [44].

S100a9 is a small calcium-binding protein that belongs to the S100 family. It is primarily expressed by neutrophils but also by activated monocytes and macrophages [45]. S100a9 has been shown to have a chemoattractant function in inflamed tissue, attracting neutrophils and inducing the adhesion of the attracted cells [46]. Elevated levels of S100a9 have been found in the synovial fluid [47] and plasma [48] of patients with RA.

### Identification of interferon-regulated pathways

To find a connection between the differentially expressed genes bioinformatic tools (Ingenuity Pathway Analysis; Ingenuity systems Inc., CA, USA, and PathwayAssist; Stratagene, CA, USA) and literature studies were used to assign pathways for these genes. Using these analysis tools, large schematic diagrams, covering all published interactions between the identified genes and common denominators, are produced. However, in this study we chose to focus on and validate one common denominator for the regulation of the identified differentially expressed genes, i.e.*Ifn *types I and type II (*Ifn*γ) [35,49]. In addition, this stratification of the bioinformatic information was performed to make it possible to verify these findings using quantitative real-time PCR and to validate the biological importance of the identified pathway in further biological experiments. Hence, the identified central pathways for further studies included the genes increased in expression due to treatment with pristane (*Ass, Cxcl9 and Mmp12*), which are regulated by Ifn-γ and genes with increased expression due to phytol (*Best5, Irf7, Ifit3, Oas1, Mx2 and S100a9*), which are induced by IFN-α/β.

Both IFN-α/β and IFN-γ have an important role in the immunological response to pathogens and viruses. However, type I IFNs are mainly produced by plasmacytoid dendritic cells [50], whereas IFN-γ is produced by macrophages, natural killer (NK) cells and activated T helper (Th) cells. Consequently, the *Ifn *genes were included in the panel of genes to be further studied.

### Time study

**Figure 3:**
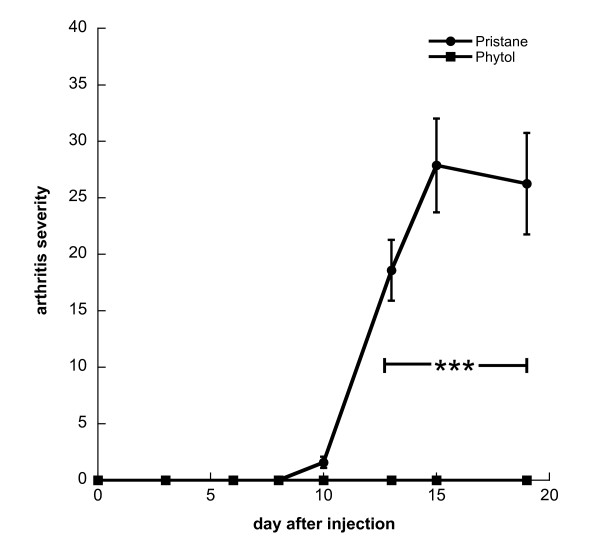
**Arthritis development in rats after subcutaneous administration of pristane (circles) or phytol (squares) in the time study experiment**. Only animals injected with pristane developed arthritis. Values are means ± SEM from groups of four animals. Levels of significance were calculated using Student's *t*-test (***p < 0.001).

To analyze how the expression of the selected genes (Table 1) varies over time, a third biological experiment was performed. In this experiment, only pristane-treated or phyto- treated animals were studied, and inguinal lymph nodes were isolated at 0, 3, 6, 8, 10, 13, 15 and 19 days after injection. This time study enabled us to follow the expression of these genes during the development of arthritis induced by pristane (Figure 3).

**Figure 4:**
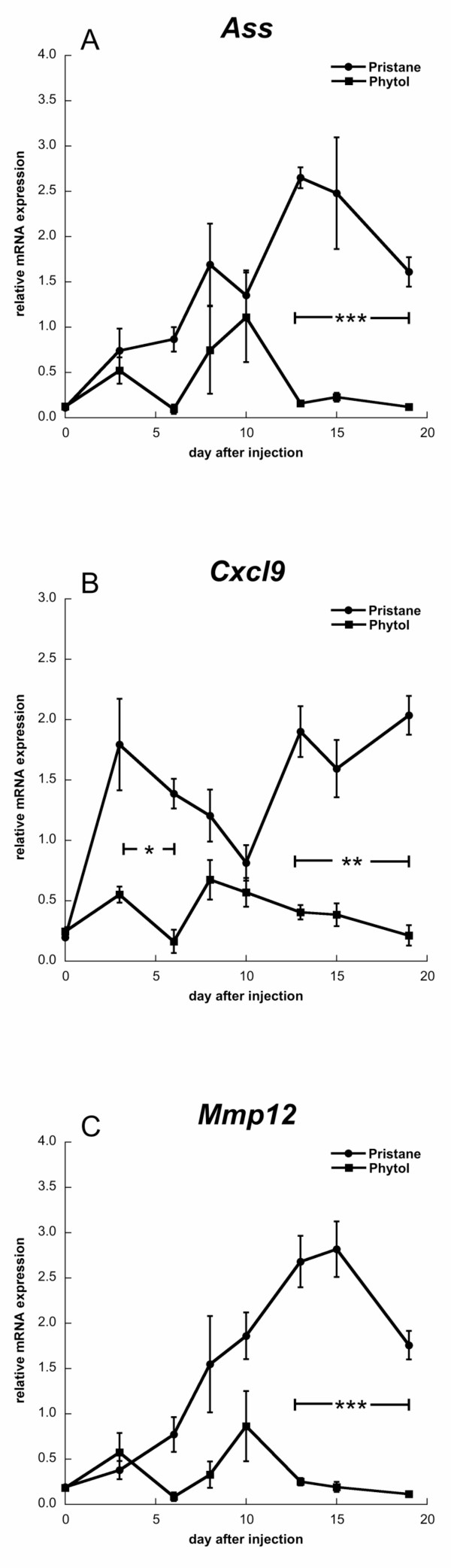
**The relative mRNA expression of (A) *Ass*, (B) *Cxcl9 *and (C) *Mmp12 *in lymph nodes at 0, 3, 6, 8, 10, 13, 15 and 19 days after injection with pristane (circles) or phytol (squares)**. Values are means ± SEM from groups of four animals. Levels of significance were calculated using Student's *t*-test (*p < 0.05; **p < 0.01; ***p < 0.001).

The three genes associated with increased expression after pristane administration (*Ass*, *Cxcl9 *and *Mmp12*) showed significantly increased expression (*p *< 0.01) after disease onset (i.e. days 13–19 after pristane administration) compared with the phytol-treated group. Phytol induced a small increase in expression of these genes around the day of disease onset (day 8–13) and then decreased to the same low expression as seen at day 0 (Figure 4). The fact that the highest expression of these genes occurs after arthritis onset makes it plausible that the increased expression of these genes is associated with the inflammatory response. However, as seen for the expression of *Cxcl9 *(Figure 4B), which also increased significantly (*p *< 0.05) at days 3–6 after pristane injection, the expression of such genes also plays a role in the early response after injection.

**Figure 5:**
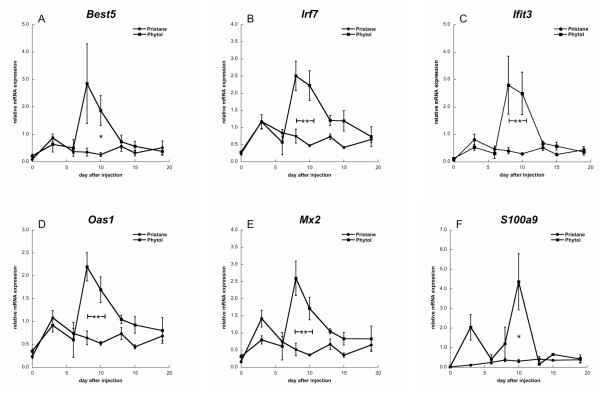
**The relative mRNA expression of (A) *Best5*, (B) *Irf7*, (C) *Ifit3*, (D) *Oas1*, (E) *Mx2 *and (F) *S100a9 *in lymph nodes at 0, 3, 6, 8, 10, 13, 15 and 19 days after injection with pristane (circles) or phytol (squares)**. Values are means ± SEM from groups of four animals. Levels of significance were calculated using Student's *t*-test (*p < 0.05; **p < 0.01).

All genes associated with increased expression after phytol administration (*Best5*, *Irf7*, *Ifit3*, *Oas1*, *Mx2 *and *S100a9*) showed significantly increased expression (*p *< 0.05) compared with pristane (Figure 5) in the early disease-onset period (day 8–10). The expression levels then decreased to the same low level as for the pristane-treated rats.

Besides the genes described above, *S100a8 *and the *Mx2*-related gene *Mx1 *were also analyzed in this time study and shown to be expressed in a similar pattern to that of *S100a9 *and *Mx2*, respectively (data not shown). As these genes are closely related, only one of each was followed for further expression characterization. The expression of *Ncf1 *was also included in this analysis as a control. However, no marked difference in *Ncf1 *expression was detected between the groups of animals (data not shown).

**Figure 6:**
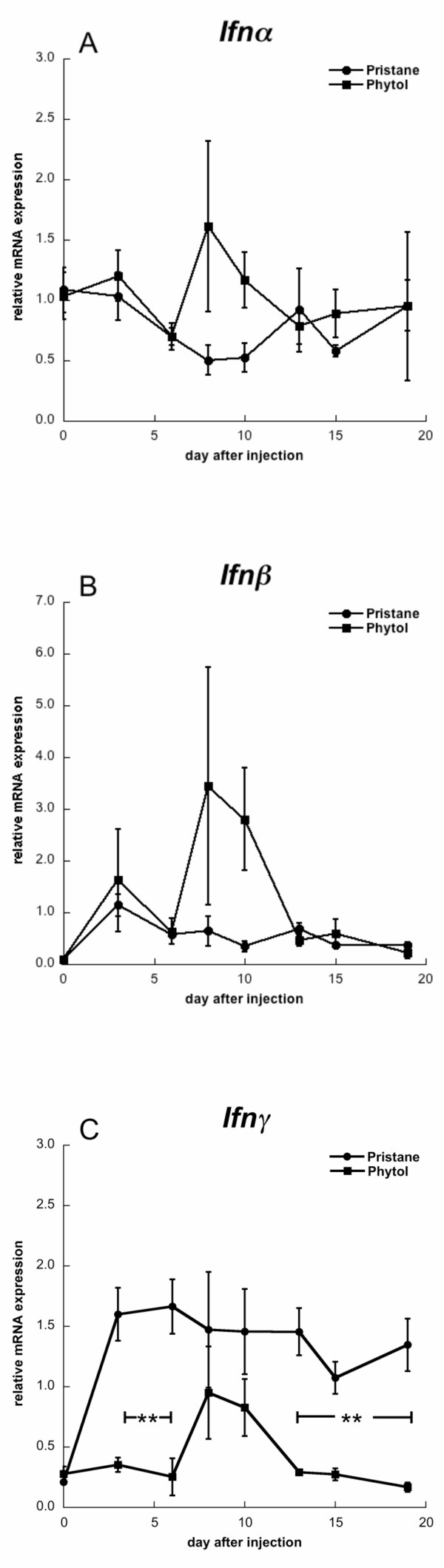
**The relative mRNA expression of (A) *Ifn*α, (B) *Ifnβ *and (C) *Ifnγ *in lymph nodes at 0, 3, 6, 8, 10, 13, 15 and 19 days after injection with pristane (circles) or phytol (squares)**. Values are means ± SEM from groups of four animals. Levels of significance were calculated using Student's *t*-test (**p < 0.01).

The expressions of the *Ifn *genes were also analyzed over time (Figure 6). The expression of *Ifnγ *was significantly increased in the rats injected with pristane (*p *< 0.01) and expression of *Ifnα *and *Ifn*β was increased after injection with phytol. To further validate the significance of the increased expression of *Ifnα *and *Ifnβ *in DA rats at day 10 after treatment with phytol, RNA samples produced from all four separate experiments was analyzed for *Ifnα *and *Ifnβ *expression levels in DA rats treated with either pristane (*n *= 19) or phytol (*n *= 18). This combined dataset showed an upregulation of IFN-α (*p *< 0.05) and an upregulation of IFN-β (*p *< 0.01) in phytol-treated compared with pristane-treated animals. The increased expression of *Ifn*α/β coincided with the most pronounced increase in expression of *Best5, Irf7, Ifit3, Oas1, Mx2 *and *S100a9*, i.e. day 8–10 after treatment (Figure 5 and Figure 6A, B). Hence, *Ifnα *and *Ifnβ *could be postulated to be common regulatory genes for this molecular pathway of genes that were all induced by phytol treatment. However, it should be noted that the expression levels of the studied type I interferons were very low and that the difference in expression levels could only be detected using quantitative real-time PCR (Figures 6 and 9).

TNF-α and IL-1 are other cytokines identified as important immunological regulators in the same molecular pathways as the interferons. Transcription of *Tnfα *and *IL-1 *is increased in macrophages activated by IFN-α to act synergistically with IFN-γ in initiating a chronic inflammatory response. The expression of *Tnfα *and *IL-1 *was also analyzed, but only a small decrease in the expression of *Tnfα *could be observed after phytol administration, and the expression of *IL-1 *was below the detection limit (data not shown).

### Verification of *Ifn *type I pathway in *Ncf1 *congenic rats

**Figure 7:**
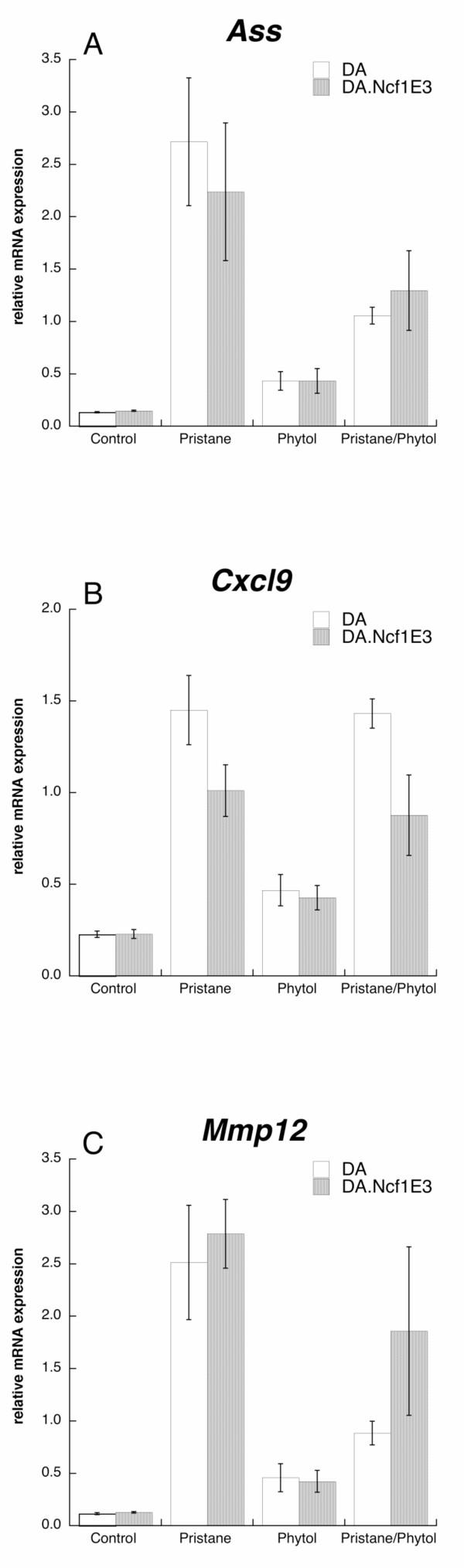
**Gene-expression comparison between DA (open bars) and DA. *Ncf1*^E3 ^(filled bars) of (A) *Ass*, (B) *Cxcl9*, and (C) *Mmp12 *in inguinal lymph nodes 10 days after injection**. Values are means ± SEM of 4–5 animals per group. No significant difference between the strains was detected.

The selected genes that were identified as being associated with either the disease state (pristane) or treatment state (phytol) of DA rats, were further analyzed in another biological experiment including a comparison between DA and congenic DA.*Ncf1*^E3 ^rats. These rats carry different alleles of the *Ncf1 *gene, and differ dramatically in arthritis severity; the DA rat is highly susceptible and the congenic DA.*Ncf1*^E3 ^is almost completely resistant to arthritis [14]. This analysis was included to study the importance of the selected genes for the difference in arthritis susceptibility caused by different *Ncf1 *alleles, and thus also NADPH oxidase functionality in PIA compared with phytol treatment. Administration with either pristane or phytol leads to increased expression of *Ass, Cxcl9 *and *Mmp-12 *in both strains, compared with the untreated controls (*p *< 0.01) (Figure 7). Further, a clear and significant difference (*p *< 0.001) between the pristane-treated and phytol-treated animals was observed, although no significant difference was found between the two rat strains.

**Figure 8:**
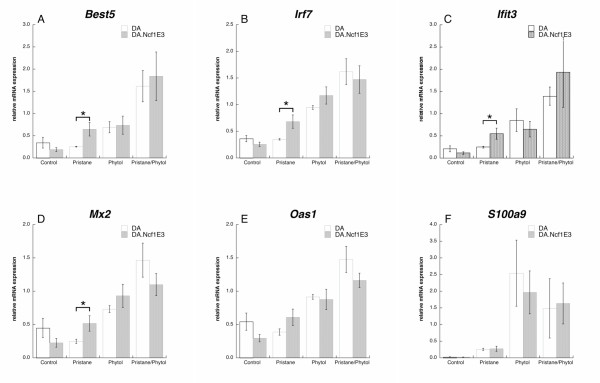
**Gene-expression comparison between DA (open bars) and DA. *Ncf1*^E3 ^(filled bars) of (A) *Best5*, (B) *Irf7*, (C) *Ifit3*, (D) *Oas1*, (E) *Mx2 *and (F) *S100a9 *in inguinal lymph nodes 10 days after injection**. Values are means ± SEM of 4–5 animals per group. Levels of significance between the strains were calculated using Student's *t*-test (*p < 0.05).

Expression of *Best5*, *Irf7*, *Ifit3*, *Mx2*, *Oas1 *and *S100a9 *were all induced by phytol, compared with untreated controls (*p *< 0.01). Interestingly, after pristane injection, there was significantly higher expression (*p *< 0.05) of *Best5, Irf7, Ifit3 *and *Mx2 *in DA.*Ncf1*^E3 ^rats than in DA rats. No difference in expression levels between the strains was observed for *S100a9*. Overall, the gene expression of *Best5, Irf7, Ifit3, Mx2 *and *Oas1 *showed the highest expression levels in rats that had been injected with a combination of pristane and phytol (Figure 8).

**Figure 9:**
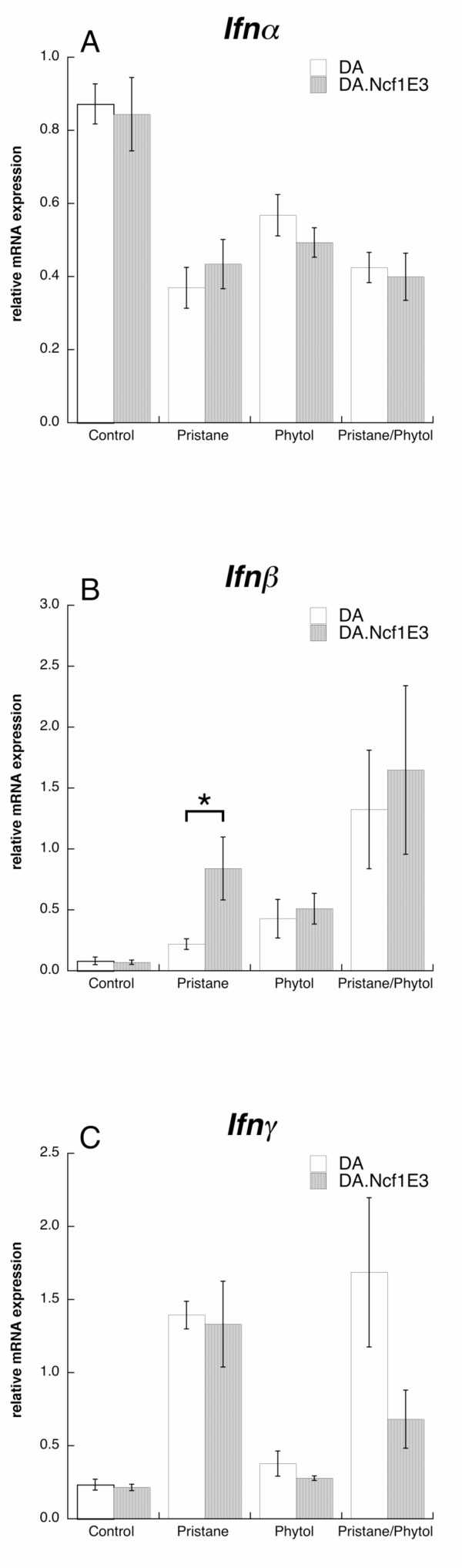
**Gene-expression comparison between DA (open bars) and DA. *Ncf1*^E3 ^(filled bars) of (A) *Ifn*α, (B) *Ifnβ *and (C) *Ifnγ *in inguinal lymph nodes 10 days after injection**. Values are means ± SEM of 4–5 animals per group. Levels of significance between the strains were calculated using Student's *t*-test (*p < 0.05).

*Ifnα *was expressed at a lower level in both the pristane-treated and phytol-treated animals, and in the rats with combined treatment, compared with the untreated control rats (*p *< 0.01) (Figure 9A). The expression of *Ifnβ *was significantly higher (*p *< 0.01) in the pristane-injected or phytol-injected groups than in the untreated control group, while the combined treatment induced even higher expression (Figure 9B). The expression of *Ifnγ *was significantly higher (*p *< 0.01) for the rats injected with pristane than for both control and phytol-treated rats (Figure 9C). Interestingly, only *Ifnβ *showed different expression in the DA.*Ncf1*^E3 ^rats compared with DA rats after pristane injection (*p *< 0.05). The expression level of *Ifnβ *in DA rats after pristane injection was comparable with that of untreated control rats; however, after phytol administration, the level of *Ifnβ *was increased to a level equal to that of DA.*Ncf1*^E3 ^rats (Figure 9B).

### Cell distribution in *Ncf1 *congenic rats

**Figure 10:**
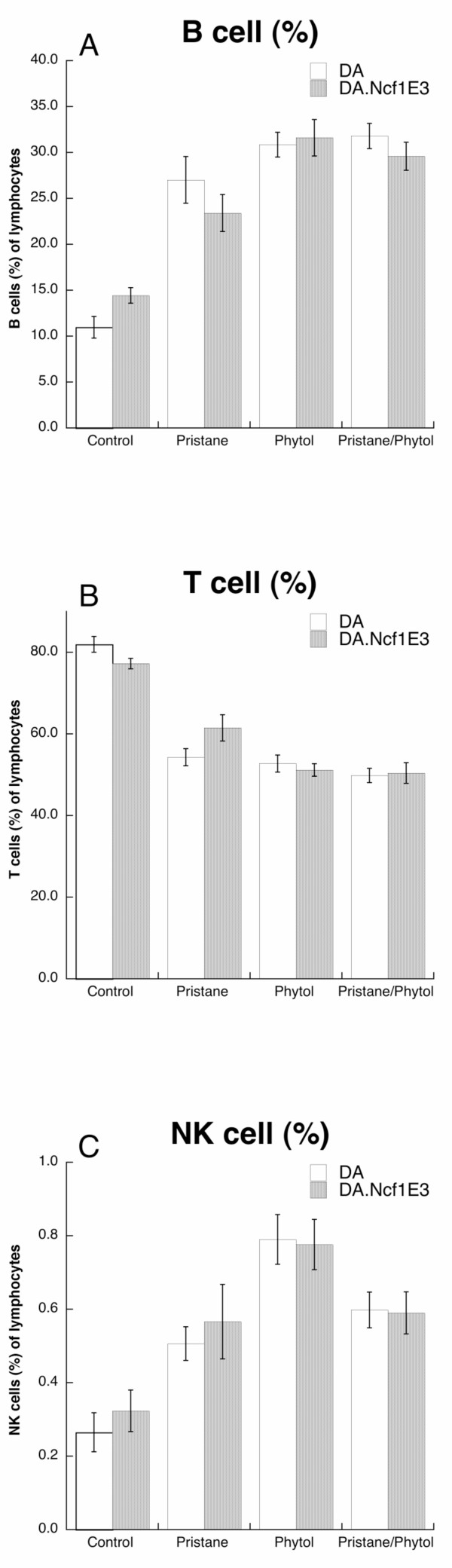
**Relative (%) number of lymphocytes**. Comparison between DA (open bars) and DA.*Ncf1*^E3 ^(filled bars) of (A) B cells, (B) T cells and (C) NK cells in inguinal lymph nodes 10 days after treatment injection. Values are means ± SEM of 4–5 animals per group. No significant difference between the strains was detected.

The levels of different cell populations in the studied tissue may affect the outcome of gene-expression profiling. Therefore, fluorescence-activated cell-sorting (flow cytometry, BD Biosciences) analyses were performed on isolated inguinal lymph nodes to determine whether the observed differentially expressed genes could be explained by a skewed cell population. In both the pristane-treated and phytol-treated rats, the number of B and NK cells were raised compared with untreated controls, whereas the relative number of T cells were lower. Significantly more B and NK cells were observed after phytol treatment than after pristane treatment (*p *< 0.01, DA and DA.Ncf1^E3 ^combined). No significant alterations in the subset of cells containing macrophages and dendritic cell populations (i.e. CD4+TCR- cells) could be detected (data not shown). No difference between the strains was observed for all of the studied groups of animals (Figure 10). These effects on cell populations could have a major impact on the gene-expression profiles, but as no difference between the strains was observed, the differentially expressed genes assigned to the *Ifnβ *pathway are unlikely to be the result of differences in these cell populations.

## Discussion

*Ncf1 *is a gene encoding the activating component of the NADPH oxidase complex. By positional cloning in rat models of arthritis, a polymorphism of *Ncf1 *was found to regulate arthritis severity [14]. In fact, it was shown that a less functional *Ncf1*, and the resulting decrease in NADPH oxidase capacity to produce ROS, is a major cause of increased arthritis severity in both rat and mouse models of arthritis, an observation that challenges the general dogma of the inflammatory role of ROS. Furthermore, NADPH-activating substances have both preventive and therapeutic effects on arthritis, which opens up new approaches for disease treatment [20]. However, from the results of the positional cloning of *Ncf1 *and the data showing strong ameliorating effects of ROS-inducing compounds in animal models, we are still a long way from fully understanding the underlying mechanism of action.

One approach to obtaining a molecular insight into a complex biological system is to use global gene-expression profiling [51]. An advantage of this method is the large set of genes that can be analyzed in the same experimental setup, making it possible to use clustering and pathway analysis of large sets of genes [52,53]. The assembled biological information can then be stratified into molecular pathways that can be studied and thoroughly validated.

However, the intricate choice of time point and tissue/cell type to be selected for analysis presents a significant hurdle [54]. A further difficulty is the need for biological replicates and the genetic heterogeneity that is involved in human studies. This has naturally led to some skepticism regarding the utility of gene-expression profiling as a method to achieve an understanding of rheumatic diseases [55]. However, recent analysis of gene-expression fingerprints of individual patients with RA might give hope for this still technically evolving method for understanding complex disorders [56-59].

In this study, we used global gene-expression analysis and quantitative real-time PCR techniques in four separate arthritis experiments in rats to investigate the downstream effects of preventive arthritis treatment with the NADPH oxidase activator phytol. Based on biodistribution analyses of phytol after SC administration, global gene-expression analysis was performed on inguinal lymph nodes at a time point just before the estimated day of disease onset. We observed that the expression of our set of differentially expressed genes in our material varies dramatically during the disease progress (Figures 4, 5, 6). Therefore, it is necessary to have information about which time point and tissue to analyze before initiating gene-expression profiling. Furthermore, we also analyzed, in other tissues (i.e. thymus, blood and spleen) the expression of the genes identified as differentially expressed in inguinal lymph nodes, and observed that the expression levels could be quite different or even undetectable in these tissues (data not shown). As a result, it is not possible to extrapolate the information between tissues and time points. In addition, the number of biological replicates used in the compared groups should be high enough to enable statistical analysis. Altogether, these issues make these studies extremely cumbersome to perform, especially when analyzing human samples. By using animal models, a more optimized study design may be provided [60], as a number of identical (inbred) individuals under the same treatment and environmental conditions are compared and tissue collected at the same time point by the same researcher.

**Figure 11:**
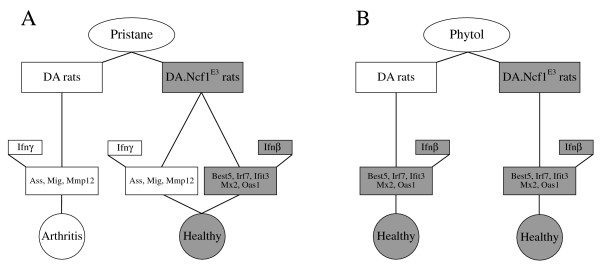
**Schematic representation of the differentially expressed genes and how they are induced upon injection by (A) pristane or (B) phytol**. The molecular pathway that is shared between the arthritis resistant DA.*Ncf1*^E3 ^rat strain and the arthritis treatment achieved with phytol is the Ifn-β-dependent pathway, which results in the upregulation of Best5, Irf7, Ifit3, Oas1 and Mx2. The inflammatory arthritis that is induced by an injection with pristane is characterized by increased expression of the *Ifn*γ-connected signature genes *Ass*, *Cxcl9 *and *Mmp12*, which potentially could be used as molecular markers for an initiating inflammatory response. As this pathway is also induced by pristane in the DA.*Ncf1*^E3 ^strain this upregulation *per se *does not cause arthritis. Most important in this mechanism is how administration of phytol induces arthritis protection via the Ifn-β-regulated pathway in the arthritis-susceptible DA rats.

In this study, we identified a molecular mechanism that links the protective effects observed from treatment with NADPH oxidase activators with a relevant inflammatory mechanism. However, it is not the differentially expressed genes *per se *that are most interesting, but the molecular pathways that can be extracted (Figure 11). From our comparison between pristane (arthritis inducer [21]) and phytol (NADPH oxidase-activating and arthritis-ameliorating compound [20]), two main pathways were identified. The pristane-induced pathway was marked by increased expression of *Ass*, *Cxcl9 *and *Mmp12 *(Table 1). These genes all share IFN-γ as a common regulator [30,61,62], which was also shown to be upregulated in the inguinal lymph nodes, in a similar pattern, after induction of arthritis with pristane (Figures 4, 6C, 7 and 9C). IFN-γ is known to effect the balance between Th1 and Th2 cells, and increased expression of this pro-inflammatory cytokine plays an important role in the progression of RA [63]. Although pristane stimulates the expression of these cytokine-regulated genes, no direct conclusions about whether these genes are involved in the cause of arthritis can be drawn, as no difference in gene expression could be seen between the arthritis-susceptible strain (DA) and the arthritis-protected strain (DA.*Ncf1*^E3^) (Figures 7 and 9C). As the DA.*Ncf1*^E3 ^congenic rats carry the arthritis-protecting variant of *Ncf1*, one might speculate that the effect caused by the functional *Ncf1 *is to generate a resistance to the effect from induction of *Ass, Cxcl9 *and *Mmp12 *genes, which might otherwise contribute to the development of arthritis. Further investigation of the effect on disease regulation is needed to be able to propose a more exact role for these genes as potential markers of arthritis [64].

Six genes (*Best5, Irf7, Ifit3, Mx2, Oas1 *and *S100a9*) were identified as genes induced by phytol (Table 1). The common inducers of these are IFN-α and IFN-β. These interferons are anti-inflammatory cytokines expressed after pathogenic infections [35,65,66]. From the gene-expression data it is not possible to determine whether IRF-7 is upregulated by an increased level of IFN-α/β as a downstream feedback regulator of the IFN-α/β receptor or if increased IFN-β level is the result of an upregulation of IRF-7 [35,41,36]. *Oas1 *and *Mx2 *are both induced upon viral invasion and IFN type I activation, and are thus regulated by the same pathway [67]. The expression of *Best5 *can be induced by both IFN-α/β and IFN-γ, but studies have shown IFN-α/β to have the strongest enhancing effect on the expression [32].

Interestingly, a clear tendency in the expression of *Ifnβ *between DA and DA.*Ncf1*^E3 ^strains was observed after injection with pristane, with the DA.*Ncf1*^E3 ^rats having higher expression of *Ifnβ *than the DA rats (Figure 9B). This difference in expression level clearly resembles the differences observed for the phytol-induced genes *Best5, Irf7, Ifit3, Mx2 *and *Oas1 *(Figure 8). It is therefore likely that the downstream effect of the polymorphism in *Ncf1*, as well as the therapeutic effect of phytol, involves regulation of an IFN-β pathway. This IFN-β-regulated gene profile is interesting with respect to similarities to the patterns that have been identified in patients with SLE and in patients with juvenile arthritis treated with anti-TNF-α [68-71], and in mouse models of SLE [72]. Subpopulations with active RA have also been shown to express increased levels of *Ifn *type I signature genes in peripheral blood [59]. These reports show an *Ifn*α/*β *expression signature, which is very similar to that identified in the rats treated with phytol. However, in our study, the *Ifn *type I expression profile was linked to disease protection while the SLE *Ifn *type I profile was linked to disease progression. This may indicate that the interferon balance could be an important threshold denominator of autoimmune diseases, functioning as an essential balance for immune regulation, with an imbalance resulting in either SLE or arthritis. This is also exemplified by a recent study in which pristane was used to induce SLE in mice. In that study, chronic peritoneal administration of pristane elicited increased expression of the type I interferon-inducible genes *Mx1*, *Irf7, IP-10 *and *Isg-15 *as a consequence of SLE [72]. However, despite the similarities in expression pattern, it must be noted that the SLE studies were performed on tissues other than inguinal lymph nodes and on established disease, so that differences in gene profiles could be caused by tissue differences or time-dependent regulation. Also interesting is the fact that the present study points towards a disease-ameliorating pathway that is regulated by IFN-β. Therefore, as the present observation of increased *Ifn *type I signature genes was observed to be a signal for prevention of disease onset, in contrast to the studies in ongoing SLE [68]and arthritis [59], one might speculate that increased IFN type I regulation is a way to downregulate an ongoing immune response. Such an attempt to limit the inflammatory response has been suggested previously [73], and has also been indicated by experiments in our laboratory using *Ifn*β-deficient mice, where a prolonged arthritis severity was observed due to *Ifnβ *deficiency [74]. In fact, treatment with recombinant IFN-β significantly reduces cartilage destruction and bone destruction in collagen-induced arthritis in mice, which suggested a beneficial effect in patients also [70]. However, to date no positive outcome of clinical trials using recombinant IFN-β in RA has been presented [75,76]. As we have shown that the effect of phytol on *Ifn*β-related genes is time-dependent and also correlates with the biodistribution of phytol to the inguinal lymph nodes, it is possible that tissue distribution as well as the dose and frequency of administration are crucial for the efficacy of arthritis treatment via this pathway.

The data presented here, together with gene-expression profiles of SLE, [68-71] and arthritis [59] strongly suggests the importance of the IFN-α/β pathway as a key mechanism in autoimmune conditions [77].

The fact that no difference in cell populations was observed to explain the differential levels of mRNA for IFN-β-regulated genes does not prove that alterations in cell populations in the inguinal lymph nodes are not crucial. As IFN-β is mainly produced by plasmacytoid dendritic cells [77], which were not specifically addressed in this study, more careful analysis of this cell population might provide further insight into the disease-protecting effects of phytol in arthritis.

Increased expression of *S100a9 *was observed in rats treated with phytol (Figure 5F). However, the expression of *S100a9 *did not show any difference between DA and DA.*Ncf1*^E3 ^rats (Figure 8F), and the expression profile was not similar to that of *Ifnβ *(Figure 9B). As a result, we consider *S100a9 *not to be regulated by IFN-β. It is more likely that the increased levels of *S100a9 *expression is a direct effect of increased concentrations of intracellular Ca^2+ ^induced by the NADPH oxidase-produced ROS [78,79]. Increased expression of *S100a9 *is mostly reported to be proinflammatory. However, this knowledge is based on analyses from tissues and blood during ongoing inflammation, while these studies analyzed inguinal lymph nodes prior to disease onset. Hence, the expression levels for different timepoints of the disease process could differ. Even so, *S100a9 *may be a good biomarker for the *in vivo *efficacy of administered phytol as an activator of NADPH oxidase specifically in lymph node tissues.

## Conclusion

By targeting the NADPH oxidase complex with activating compounds such as phytol, we highlight a new mechanism to treat autoimmune conditions such as arthritis. By extracting and verifying a relevant biological molecular mechanism from gene-expression profiling data, we also indicate a plausible relation between increased levels of ROS and an anti-inflammatory response regulated by an IFN-β pathway. The use of gene-expression profiling to compare treatments in animal models of complex diseases also points to a useful pharmacogenomic approach to extract relevant information about the mechanism of action and to identify potential molecular biomarkers to be used in future animal experiments and clinical trials.

## Methods

### Animals

DA rats used in the microarray analysis, the first verifying quantitative real-time PCR study, the time study and the biodistribution study were purchased from Harlan Netherlands, and the DA.*Ncf1*^E3 ^rats with background origin from Zentralinstitut Für Versuchstierzucht, Hannover, Germany [80,81]. All animals were kept in a climate-controlled environment with 12-hour light/dark cycles, housed in polystyrene cages containing wood shavings and fed standard rodent chow and water *ad libitum*. The rats were free from common pathogens including Sendai virus, Hantaan virus, coronavirus, reovirus, cytomegalovirus and *Mycoplasma pulmonalis*. The experiments were approved by the local ethics committees (Göteborg, Swedish license 230/2003 and Malmö/Lund, Swedish license M70/2004).

#### Induction and evaluation of arthritis

Pristane (Sigma-Aldrich, St. Louis, MO, USA) and/or phytol (3,7,11,15-tetramethyl-2-hexadecene-1-ol) (Sigma-Aldrich) were injected into the rats (age 8–12 weeks) by a single subcutaneous injection of 200 μL at the base of the tail. Arthritis development was monitored with a macroscopic scoring system of the four limbs ranging from 0 to 15 (1 point for each swollen or red toe, 1 point for midfoot digit or knuckle, 5 points for a swollen ankle). The scores of the four paws were added, yielding a maximum total score of 60 for each rat [82].

In the global gene-expression profiling and the first verifying quantitative real-time PCR experiment, five rats per group were used. In the comparative experiment between the DA and the DA.*Ncf1*^E3 ^strain, 4–5 rats per group were used. In all three experiments, the analyzed groups were; naïve controls, pristane-treated, phytol-treated and pristane plus phytol-treated animals. All rats were killed 10 days after injection. In the time-resolution study for comparison between pristane and phytol, four animals from each group were killed at 0, 3, 6, 8, 10, 13, 15 and 19 days after injection. The inguinal lymph nodes were immediately surgically removed and stored in a tissue-storage reagent (RNA *later*; Qiagen, Germany).

### Biodistribution

DA rats were injected with 200 μL phytol (Sigma-Aldrich) mixed with tritiated phytol (Moravek Biochemicals, CA, USA) to a final dose of 167 μCi/rat. The rats, four each day, were killed at 2, 4, 8, 11 or 17 days after injection, and the inguinal lymph nodes, spleen, heart, thymus, kidney, liver, lung, adipose tissue, muscle, injection-site tissue and blood were collected in equal amounts of saline solution (blood samples had heparin added to prevent coagulation) in pre-weighed tubes. The tissues were weighed, homogenized, and mixed with ready-safe scintillation liquid (Beckman Coulter, CA, USA). Tissue distribution of phytol was determined as counts per minute (cpm) of tritium using a beta counter (LKB Wallac, Turku, Finland) and cpm/g tissue was determined as the relative distribution of phytol. Microautoradiography was performed on three individual rats 10 days after subcutaneous administration with tritiated phytol (167 μCi/rat). The inguinal lymph nodes were snap-frozen in liquid nitrogen and shipped on dry ice to Quest Pharmaceutical Services (Newark, DE, USA). The frozen lymph nodes were embedded in optimum cutting temperature (OCT) embedding media (VWR international, Bristol, CT, USA) for cryosectioning. Sections of 10 μm thickness were heated at 50°C for 10 minutes, coated with photographic emulsion (Kodak NTB; Kodak, New Haven, CT, USA) and dried. The coated slides were exposed at 4°C in lightproof boxes. The exposed slides were stained with hematoxylin and eosin and developed for tritium labeling.

### Flow cytometry analysis

Single-cell suspensions were made from inguinal lymph nodes and cells were stained with the anti-rat antibodies OX-1 (anti-LCA, lymphocytes), OX-33 (anti-CD45RA, B-cells), and R73 (anti-αβTCR, T cells) (all BD Pharmingen, San Diego, CA, USA), and with 3.2.3 (anti-NKRP1, NK cells, produced from an in-house hybridoma) for 30 minutes at 4°C. After washing with phosphate-buffered saline (PBS), cells were resuspended in PBS and analyzed in a FACSorter (Becton Dickinson, San Jose, CA, USA). Gates were set for the relevant cell type and analysed as percentage of total lymphocytes.

### RNA isolation

Total RNA from inguinal lymph nodes was isolated using a commercial kit (RNeasy^® ^Mini Kit; Qiagen, Germany). The protocol for animal tissues was followed with the addition of the optional DNase digestion (RNase-Free DNase Set; Qiagen). The RNA yield was quantified spectrophotometrically (RNA 6000 Nano assay Kit;Agilent Technologies, Palo Alto, CA, USA) and the quality analyzed (2100 Bioanalyzer; Agilent). The average ratio between 28S/18S rRNA was 2.2, indicating high RNA quality. All RNA and cDNA samples were stored at -80°C.

### Preparation of cRNA, hybridization and data analysis

In total, 10 μg of total RNA spiked with poly-A controls (pGIBS-TRP, pGIBS-THR, and pGIBS-LYS; American Type Culture Collection) was converted to cDNA, using a T7 promoter-polyT primer (Affymetrix, Santa Clara, CA, USA) and the reverse transcriptase Superscript II (Invitrogen, Paisley, UK), followed by a second-strand cDNA synthesis (Invitrogen). Double- stranded cDNA was *in vitro *transcribed to biotinylated cRNA (IVT labelling kit; Affymetrix) and then fragmented. The fragmented cRNA was mixed with hybridization spike controls (oligonucleotide B2 and a cRNA cocktail: BioB, BioC, BioD, and Cre; Affymetrix,). Aliquots of each sample were hybridized (16 hours at 45°C) to an array (GeneChip^® ^Rat Expression Set 230A arrays; Affymetrix). The arrays were subsequently washed, stained and scanned according the manufacturer's instructions (GeneChip^® ^Expression Analysis Technical Manual; Affymetrix). The data were analyzed using specific sofware (Robust Multi-Chip Analysis in GeneTraffic^® ^UNO version 3.2–11; Stratagene, La Jolla, CA, USA, and Spotfire DecisionSite for Functional Genomics, version 8.1;Spotfire Inc., Göteborg, Sweden). The intensities were log_2 _transformed and the mean log_2 _intensity for each group calculated. The mean log_2 _fold change was calculated for the phytol-treated animals versus the pristane-treated animals by subtracting the mean log_2 _intensity for the pristane-treated rats from that for the phytol-treated rats. The total number of probe sets in the used rat Affymetrix chips was 15 923, and the average present call was 45%. Statistical significance of the difference in gene expression was determined using the two-sided Student's *t*-test. A transcript was considered differentially expressed if the mean absolute fold change was > 1.4 and the p value < 0.05. In addition, the mean intensity in the group showing the highest expression should be > 75. The average log_2 _fold change between the animals treated with phytol plus pristane versus the animals treated with pristane alone and the corresponding statistical analysis were also calculated, although these data were not used to identify differentially expressed genes.

### Accession numbers

The global gene-expression profiling data is deposited on-line [ArrayExpress: E-MEXP-78].

### Quantitative real-time PCR

The primers used in the quantitative real-time PCR were designed using software (Primer Express 2.1; Applied Biosystems, Foster City, CA, USA) and the sequences are listed in Table 2. Total RNA (5 μg) was transcribed to cDNA using a commercial system (SuperScript™ First Strand Synthesis System; Invitrogen). The PCR reaction was performed in a 25 μL volume including 1 × SYBR^® ^Green (Applied Biosystems) and 400 nM of each primer, except for *Inf*β, for which 800 nM of each primer was used. All reactions were performed in duplicate, amplified and quantified (ABI 7000 Sequence Detection System; Applied Biosystems). The relative quantities of mRNA were calculated according to the standard curve method [83] and *Arbp (alias 36B4)*, was used as endogenous control [84].

### Statistics

Quantitative data is expressed as mean ± SEM and significance analysis was performed using two-sided Student's T-test. * Represents a significance value of * *p *< 0.05, ** *p *< 0.01 and *** *p *< 0.001.

## Authors' contributions

PO, AN, MH and EN contributed to the design and performed the experiments, collected and interpreted the data, and wrote the manuscript. SA and AB performed the real-time PCR under supervision of AN and PO. RH contributed to the study design, interpretation of collected data and the writing of the paper.

## Accession numbers

The global gene-expression profiling data is deposited at ArrayExpress (accession number E-MEXP-78).

## Figures and Tables

**Table 1:** Differentially expressed genes selected for further studies of the mechanism involved in pristane-induced arthritis and treatment with phytol.

Gene name	UniGene ID number	Protein	Fold change*	*p*
*Best5*	Rn.14882	Bone-expressed sequence tag 5	2.17	0.005
*Irf7*	Rn.101159	Interferon regulatory factor 7	1.75	0.005
*Ifit3*	Rn.35775	Interferon-induced protein with tetratricopeptide repeats 3	2.22	0.008
*Oas1*	Rn.10383	2',5'-oligoadenylate synthetase 1	1.69	0.01
*Mx2*	Rn.10374	Myxovirus (influenza virus) resistance 2	1.70	0.02
*S100a9*	Rn.6703	Calcium binding protein a9	3.60	0.007
*Ass*	Rn.5078	Argininosuccinate synthetase	- 3.24	0.0009
*Mmp12*	Rn.33193	Matrix metalloproteinase 12	- 3.82	0.0007
*Cxcl9*	Rn.7391	Chemokine (C-X-C motif) ligand 9	- 1.86	0.004

The genes were chosen based on the difference in expression levels between phytol and pristane treatment in the Affymetrix Genechip^® ^analysis.

*Phytol versus pristane.

**Table 2:** Primers used for the quantitative real-time PCR.

UniGene	Gene name	FP/RP	Sequence (5'-3')
Rn.1079	Arbp	FP	AGAGTCGGAGGAATCCGATGA
		RP	AAGCAGGCTGACTTGGTGTGA
Rn.38575	*Ncf1*	FP	CGAGAGTGGCTGGTGGTT
		RP	CATACGGTTCACCTGCGTAGT
Rn.5078	*Ass*	FP	TGTTCGCCACTGCATCGA
		RP	TGGCCCTTGAAGACAGATACCT
Rn.7391	*Cxcl9*	FP	ACGGAGATCAAACCTGCCTAGAC
		RP	CATCAGCTTCTTCACCCTTGCT
Rn.33193	*Mmp12*	FP	AGGTACGATGTGAGGCAGGAA
		RP	TCCTGGGAAGTGTGTAGAAATCAG
Rn.14882	*Best5*	FP	TCATTCTGCGTTGGAGTTTGG
		RP	CTGAATTATTGATGGGAGGAGTCA
Rn.101159	*IRF7*	FP	CAGAACCCCAACTTCCAATAGAGT
		RP	TAGAGATGACATATAGCCAAGGAATAAGC
Rn.35775	*Ifit3*	FP	TGGGAGGCAACACGATTTG
		RP	TGACACACTTCCGGTTGTTATCAG
Rn.10383	*Oas1*	FP	GGATCACAGTCCATGCTTCTGA
		RP	CTGTGATTGGACAGTATTCAAACATG
Rn.10374	*Mx2*	FP	GTGACACCAGTGAGAAGAGGAGATT
		RP	GCTAGCCTGCGCTGAGCTT
Rn.6703	*S100a9*	FP	TGGACACAAACCAGGACAATCA
		RP	CTCATGACAGGCAAAGATCAACTT
Rn.143194	*Ifnα *1	FP	GCAACCCTCCTAGACTCATTCTG
		RP	ACCCCTACCTGCTGCATCAG
Rn.138105	*Ifnβ *1	FP	CTTGGGTGACATCCACGACTAC
		RP	GGCATAGCTGTTGTACTTCTTGTCTT
Rn.10795	*Ifn*γ	FP	AATCGCACCTGATCACTAACTTCTT
		RP	TGGGTTGTTCACCTCGAACTT

The final concentration of each primer in the sample was 400 nM, except for *Ifnβ *1, for which 800 nM was used.

FP, forward primer; RP, reverse primer.

## References

[B1] Firestein GS (2003). Evolving concepts of rheumatoid arthritis. Nature.

[B2] Gabriel SE (2001). The epidemiology of rheumatoid arthritis. Rheum Dis Clin North Am.

[B3] Symmons DP, Bankhead CR, Harrison BJ, Brennan P, Barrett EM, Scott DG, Silman AJ (1997). Blood transfusion, smoking, and obesity as risk factors for the development of rheumatoid arthritis: results from a primary care-based incident case-control study in Norfolk, England. Arthritis and rheumatism.

[B4] MacGregor AJ, Snieder H, Rigby AS, Koskenvuo M, Kaprio J, Aho K, Silman AJ (2000). Characterizing the quantitative genetic contribution to rheumatoid arthritis using data from twins. Arthritis and rheumatism.

[B5] Bannwarth B, Labat L, Moride Y, Schaeverbeke T (1994). Methotrexate in rheumatoid arthritis. An update. Drugs.

[B6] Pincus T, Marcum SB, Callahan LF (1992). Longterm drug therapy for rheumatoid arthritis in seven rheumatology private practices: II. Second line drugs and prednisone. J Rheumatol.

[B7] Feldmann M (2002). Development of anti-TNF therapy for rheumatoid arthritis. Nat Rev Immunol.

[B8] Ruderman EM, Pope RM (2005). The evolving clinical profile of abatacept (CTLA4-Ig): a novel co-stimulatory modulator for the treatment of rheumatoid arthritis. Arthritis Res Ther.

[B9] Lipsky PE, van der Heijde DM, St Clair EW, Furst DE, Breedveld FC, Kalden JR, Smolen JS, Weisman M, Emery P, Feldmann M (2000). Infliximab and methotrexate in the treatment of rheumatoid arthritis. Anti-Tumor Necrosis Factor Trial in Rheumatoid Arthritis with Concomitant Therapy Study Group. N Engl J Med.

[B10] Weinblatt ME, Kremer JM, Bankhurst AD, Bulpitt KJ, Fleischmann RM, Fox RI, Jackson CG, Lange M, Burge DJ (1999). A trial of etanercept, a recombinant tumor necrosis factor receptor:Fc fusion protein, in patients with rheumatoid arthritis receiving methotrexate. N Engl J Med.

[B11] Begovich AB, Carlton VE, Honigberg LA, Schrodi SJ, Chokkalingam AP, Alexander HC, Ardlie KG, Huang Q, Smith AM, Spoerke JM (2004). A missense single-nucleotide polymorphism in a gene encoding a protein tyrosine phosphatase (PTPN22) is associated with rheumatoid arthritis. Am J Hum Genet.

[B12] Suzuki A, Yamada R, Chang X, Tokuhiro S, Sawada T, Suzuki M, Nagasaki M, Nakayama-Hamada M, Kawaida R, Ono M (2003). Functional haplotypes of PADI4, encoding citrullinating enzyme peptidylarginine deiminase 4, are associated with rheumatoid arthritis. Nat Genet.

[B13] Tokuhiro S, Yamada R, Chang X, Suzuki A, Kochi Y, Sawada T, Suzuki M, Nagasaki M, Ohtsuki M, Ono M (2003). An intronic SNP in a RUNX1 binding site of SLC22A4, encoding an organic cation transporter, is associated with rheumatoid arthritis. Nat Genet.

[B14] Olofsson P, Holmberg J, Tordsson J, Lu S, Akerstrom B, Holmdahl R (2003). Positional identification of Ncf1 as a gene that regulates arthritis severity in rats. Nat Genet.

[B15] Ueda H, Howson JM, Esposito L, Heward J, Snook H, Chamberlain G, Rainbow DB, Hunter KM, Smith AN, Di Genova G (2003). Association of the T-cell regulatory gene CTLA4 with susceptibility to autoimmune disease. Nature.

[B16] Vingsbo-Lundberg C, Nordquist N, Olofsson P, Sundvall M, Saxne T, Pettersson U, Holmdahl R (1998). Genetic control of arthritis onset, severity and chronicity in a model for rheumatoid arthritis in rats. Nat Genet.

[B17] Nauseef WM (2004). Assembly of the phagocyte NADPH oxidase. Histochem Cell Biol.

[B18] Gelderman KA, Hultqvist M, Holmberg J, Olofsson P, Holmdahl R (2006). T cell surface redox levels determine T cell reactivity and arthritis susceptibility. Proceedings of the National Academy of Sciences of the United States of America.

[B19] Hultqvist M, Holmdahl R (2005). Ncf1 (p47phox) polymorphism determines oxidative burst and the severity of arthritis in rats and mice. Cell Immunol.

[B20] Hultqvist M, Olofsson P, Gelderman KA, Holmberg J, Holmdahl R (2006). A New Arthritis Therapy with Oxidative Burst Inducers. PLoS Med.

[B21] Vingsbo C, Sahlstrand P, Brun JG, Jonsson R, Saxne T, Holmdahl R (1996). Pristane-induced arthritis in rats: a new model for rheumatoid arthritis with a chronic disease course influenced by both major histocompatibility complex and non-major histocompatibility complex genes. Am J Pathol.

[B22] Morey JS, Ryan JC, Van Dolah FM (2006). Microarray validation: factors influencing correlation between oligonucleotide microarrays and real-time PCR. Biological procedures online.

[B23] Husson A, Brasse-Lagnel C, Fairand A, Renouf S, Lavoinne A (2003). Argininosuccinate synthetase from the urea cycle to the citrulline-NO cycle. Eur J Biochem.

[B24] Nagasaki A, Gotoh T, Takeya M, Yu Y, Takiguchi M, Matsuzaki H, Takatsuki K, Mori M (1996). Coinduction of nitric oxide synthase, argininosuccinate synthetase, and argininosuccinate lyase in lipopolysaccharide-treated rats. RNA blot, immunoblot, and immunohistochemical analyses. J Biol Chem.

[B25] Liao F, Rabin RL, Yannelli JR, Koniaris LG, Vanguri P, Farber JM (1995). Human Mig chemokine: biochemical and functional characterization. J Exp Med.

[B26] Gasperini S, Marchi M, Calzetti F, Laudanna C, Vicentini L, Olsen H, Murphy M, Liao F, Farber J, Cassatella MA (1999). Gene expression and production of the monokine induced by IFN-gamma (MIG), IFN-inducible T cell alpha chemoattractant (I-TAC), and IFN-gamma-inducible protein-10 (IP-10) chemokines by human neutrophils. J Immunol.

[B27] Patel DD, Zachariah JP, Whichard LP (2001). CXCR3 and CCR5 ligands in rheumatoid arthritis synovium. Clin Immunol.

[B28] Brinckerhoff CE, Matrisian LM (2002). Matrix metalloproteinases: a tail of a frog that became a prince. Nat Rev Mol Cell Biol.

[B29] Feinberg MW, Jain MK, Werner F, Sibinga NE, Wiesel P, Wang H, Topper JN, Perrella MA, Lee ME (2000). Transforming growth factor-beta 1 inhibits cytokine-mediated induction of human metalloelastase in macrophages. J Biol Chem.

[B30] Wang Z, Zheng T, Zhu Z, Homer RJ, Riese RJ, Chapman HA, Shapiro SD, Elias JA (2000). Interferon gamma induction of pulmonary emphysema in the adult murine lung. J Exp Med.

[B31] Leppert D, Lindberg RL, Kappos L, Leib SL (2001). Matrix metalloproteinases: multifunctional effectors of inflammation in multiple sclerosis and bacterial meningitis. Brain Res Brain Res Rev.

[B32] Grewal TS, Genever PG, Brabbs AC, Birch M, Skerry TM (2000). Best5: a novel interferon-inducible gene expressed during bone formation. Faseb J.

[B33] Au WC, Moore PA, LaFleur DW, Tombal B, Pitha PM (1998). Characterization of the interferon regulatory factor-7 and its potential role in the transcription activation of interferon A genes. J Biol Chem.

[B34] Zhang L, Pagano JS (1997). IRF-7, a new interferon regulatory factor associated with Epstein-Barr virus latency. Mol Cell Biol.

[B35] Taniguchi T, Takaoka A (2002). The interferon-alpha/beta system in antiviral responses: a multimodal machinery of gene regulation by the IRF family of transcription factors. Curr Opin Immunol.

[B36] Honda K, Yanai H, Negishi H, Asagiri M, Sato M, Mizutani T, Shimada N, Ohba Y, Takaoka A, Yoshida N (2005). IRF-7 is the master regulator of type-I interferon-dependent immune responses. Nature.

[B37] Graham RR, Kozyrev SV, Baechler EC, Reddy MV, Plenge RM, Bauer JW, Ortmann WA, Koeuth T, Escribano MF, Collaborative Groups TA (2006). A common haplotype of interferon regulatory factor 5 (IRF5) regulates splicing and expression and is associated with increased risk of systemic lupus erythematosus. Nat Genet.

[B38] Sigurdsson S, Nordmark G, Goring HH, Lindroos K, Wiman AC, Sturfelt G, Jonsen A, Rantapaa-Dahlqvist S, Moller B, Kere J (2005). Polymorphisms in the tyrosine kinase 2 and interferon regulatory factor 5 genes are associated with systemic lupus erythematosus. Am J Hum Genet.

[B39] Levy D, Larner A, Chaudhuri A, Babiss LE, Darnell JE (1986). Interferon-stimulated transcription: isolation of an inducible gene and identification of its regulatory region. Proceedings of the National Academy of Sciences of the United States of America.

[B40] de Veer MJ, Sim H, Whisstock JC, Devenish RJ, Ralph SJ (1998). IFI60/ISG60/IFIT4, a new member of the human IFI54/IFIT2 family of interferon-stimulated genes. Genomics.

[B41] Sato M, Suemori H, Hata N, Asagiri M, Ogasawara K, Nakao K, Nakaya T, Katsuki M, Noguchi S, Tanaka N (2000). Distinct and essential roles of transcription factors IRF-3 and IRF-7 in response to viruses for IFN-alpha/beta gene induction. Immunity.

[B42] Witt PL, Marie I, Robert N, Irizarry A, Borden EC, Hovanessian AG (1993). Isoforms p69 and p100 of 2',5'-oligoadenylate synthetase induced differentially by interferons in vivo and in vitro. J Interferon Res.

[B43] Melen K, Keskinen P, Ronni T, Sareneva T, Lounatmaa K, Julkunen I (1996). Human MxB protein, an interferon-alpha-inducible GTPase, contains a nuclear targeting signal and is localized in the heterochromatin region beneath the nuclear envelope. J Biol Chem.

[B44] Asano A, Jin HK, Watanabe T (2003). Mouse Mx2 gene: organization, mRNA expression and the role of the interferon-response promoter in its regulation. Gene.

[B45] Kerkhoff C, Klempt M, Sorg C (1998). Novel insights into structure and function of MRP8 (S100A8) and MRP14 (S100A9). Biochim Biophys Acta.

[B46] Ryckman C, Vandal K, Rouleau P, Talbot M, Tessier PA (2003). Proinflammatory activities of S100: proteins S100A8, S100A9, and S100A8/A9 induce neutrophil chemotaxis and adhesion. J Immunol.

[B47] Berntzen HB, Olmez U, Fagerhol MK, Munthe E (1991). The leukocyte protein L1 in plasma and synovial fluid from patients with rheumatoid arthritis and osteoarthritis. Scand J Rheumatol.

[B48] Brun JG, Jonsson R, Haga HJ (1994). Measurement of plasma calprotectin as an indicator of arthritis and disease activity in patients with inflammatory rheumatic diseases. J Rheumatol.

[B49] Baccala R, Kono DH, Theofilopoulos AN (2005). Interferons as pathogenic effectors in autoimmunity. Immunol Rev.

[B50] Colonna M, Krug A, Cella M (2002). Interferon-producing cells: on the front line in immune responses against pathogens. Curr Opin Immunol.

[B51] Haupl T, Krenn V, Stuhlmuller B, Radbruch A, Burmester GR (2004). Perspectives and limitations of gene expression profiling in rheumatology: new molecular strategies. Arthritis Res Ther.

[B52] Devauchelle V, Marion S, Cagnard N, Mistou S, Falgarone G, Breban M, Letourneur F, Pitaval A, Alibert O, Lucchesi C (2004). DNA microarray allows molecular profiling of rheumatoid arthritis and identification of pathophysiological targets. Genes Immun.

[B53] Heller RA, Schena M, Chai A, Shalon D, Bedilion T, Gilmore J, Woolley DE, Davis RW (1997). Discovery and analysis of inflammatory disease-related genes using cDNA microarrays. Proceedings of the National Academy of Sciences of the United States of America.

[B54] Oertelt S, Selmi C, Invernizzi P, Podda M, Gershwin ME (2005). Genes and goals: an approach to microarray analysis in autoimmunity. Autoimmun Rev.

[B55] Lanchbury J, Hall M, Steer S (2002). Progress and problems in defining susceptibility genes for rheumatic diseases. Rheumatology (Oxford).

[B56] Batliwalla FM, Baechler EC, Xiao X, Li W, Balasubramanian S, Khalili H, Damle A, Ortmann WA, Perrone A, Kantor AB (2005). Peripheral blood gene expression profiling in rheumatoid arthritis. Genes Immun.

[B57] Olsen N, Sokka T, Seehorn CL, Kraft B, Maas K, Moore J, Aune TM (2004). A gene expression signature for recent onset rheumatoid arthritis in peripheral blood mononuclear cells. Ann Rheum Dis.

[B58] Shou J, Bull CM, Li L, Qian HR, Wei T, Luo S, Perkins D, Solenberg PJ, Tan SL, Chen XY (2006). Identification of blood biomarkers of rheumatoid arthritis by transcript profiling of peripheral blood mononuclear cells from the rat collagen-induced arthritis model. Arthritis Res Ther.

[B59] van der Pouw Kraan TC, Wijbrandts CA, van Baarsen LG, Voskuyl AE, Rustenburg F, Baggen JM, Ibrahim SM, Fero M, Dijkmans BA, Tak PP (2007). Rheumatoid Arthritis subtypes identified by genomic profiling of peripheral blood cells: Assignment of a type I interferon signature in a subpopulation of patients. Ann Rheum Dis.

[B60] Jirholt J, Lindqvist AB, Holmdahl R (2001). The genetics of rheumatoid arthritis and the need for animal models to find and understand the underlying genes. Arthritis Res.

[B61] Farber JM (1990). A macrophage mRNA selectively induced by gamma-interferon encodes a member of the platelet factor 4 family of cytokines. Proceedings of the National Academy of Sciences of the United States of America.

[B62] Flodstrom M, Niemann A, Bedoya FJ, Morris SM, Eizirik DL (1995). Expression of the citrulline-nitric oxide cycle in rodent and human pancreatic beta-cells: induction of argininosuccinate synthetase by cytokines. Endocrinology.

[B63] Feldmann M, Brennan FM, Maini RN (1996). Role of cytokines in rheumatoid arthritis. Annu Rev Immunol.

[B64] Bailey WJ, Ulrich R (2004). Molecular profiling approaches for identifying novel biomarkers. Expert Opin Drug Saf.

[B65] Decker T, Stockinger S, Karaghiosoff M, Muller M, Kovarik P (2002). IFNs and STATs in innate immunity to microorganisms. J Clin Invest.

[B66] Nguyen H, Hiscott J, Pitha PM (1997). The growing family of interferon regulatory factors. Cytokine Growth Factor Rev.

[B67] Stark GR, Kerr IM, Williams BR, Silverman RH, Schreiber RD (1998). How cells respond to interferons. Annu Rev Biochem.

[B68] Baechler EC, Batliwalla FM, Karypis G, Gaffney PM, Ortmann WA, Espe KJ, Shark KB, Grande WJ, Hughes KM, Kapur V (2003). Interferon-inducible gene expression signature in peripheral blood cells of patients with severe lupus. Proceedings of the National Academy of Sciences of the United States of America.

[B69] Baechler EC, Gregersen PK, Behrens TW (2004). The emerging role of interferon in human systemic lupus erythematosus. Curr Opin Immunol.

[B70] Bennett L, Palucka AK, Arce E, Cantrell V, Borvak J, Banchereau J, Pascual V (2003). Interferon and granulopoiesis signatures in systemic lupus erythematosus blood. J Exp Med.

[B71] Palucka AK, Blanck JP, Bennett L, Pascual V, Banchereau J (2005). Cross-regulation of TNF and IFN-alpha in autoimmune diseases. Proceedings of the National Academy of Sciences of the United States of America.

[B72] Nacionales DC, Kelly KM, Lee PY, Zhuang H, Li Y, Weinstein JS, Sobel E, Kuroda Y, Akaogi J, Satoh M (2006). Type I interferon production by tertiary lymphoid tissue developing in response to 2,6,10,14-tetramethyl-pentadecane (pristane). Am J Pathol.

[B73] Tak PP (2004). IFN-beta in rheumatoid arthritis. Front Biosci.

[B74] Treschow AP, Teige I, Nandakumar KS, Holmdahl R, Issazadeh-Navikas S (2005). Stromal cells and osteoclasts are responsible for exacerbated collagen-induced arthritis in interferon-beta-deficient mice. Arthritis and rheumatism.

[B75] van Holten J, Pavelka K, Vencovsky J, Stahl H, Rozman B, Genovese M, Kivitz AJ, Alvaro J, Nuki G, Furst DE (2005). A multicentre, randomised, double blind, placebo controlled phase II study of subcutaneous interferon beta-1a in the treatment of patients with active rheumatoid arthritis. Ann Rheum Dis.

[B76] Tak PP, Hart BA, Kraan MC, Jonker M, Smeets TJ, Breedveld FC (1999). The effects of interferon beta treatment on arthritis. Rheumatology (Oxford).

[B77] Biron CA (2001). Interferons alpha and beta as immune regulators – a new look. Immunity.

[B78] Brechard S, Bueb JL, Tschirhart EJ (2005). Interleukin-8 primes oxidative burst in neutrophil-like HL-60 through changes in cytosolic calcium. Cell Calcium.

[B79] Foell D, Frosch M, Sorg C, Roth J (2004). Phagocyte-specific calcium-binding S100 proteins as clinical laboratory markers of inflammation. Clin Chim Acta.

[B80] Olofsson P, Holmberg J, Pettersson U, Holmdahl R (2003). Identification and isolation of dominant susceptibility loci for pristane-induced arthritis. J Immunol.

[B81] Olofsson P, Holmdahl R (2003). Positional cloning of Ncf1 – a piece in the puzzle of arthritis genetics. Scand J Immunol.

[B82] Holmdahl R, Carlsén S, Mikulowska A, Vestberg M, Brunsberg U, Hansson A, Sundvall M, Larsson L, Pettersson U, Adolph KW (1998). Genetic analysis of mouse models for rheumatois arthritis. Human Genome Methods.

[B83] AppliedBiosystems (1997). User Bulletin #2 ABI PRISM 7700 sequence detection system.

[B84] Laborda J (1991). 36B4 cDNA used as an estradiol-independent mRNA control is the cDNA for human acidic ribosomal phosphoprotein PO. Nucleic Acids Res.

